# Optimization of the analogue-sensitive Cdc2/Cdk1 mutant by *in vivo* selection eliminates physiological limitations to its use in cell cycle analysis

**DOI:** 10.1098/rsob.140063

**Published:** 2014-07-02

**Authors:** Yuki Aoi, Shigehiro A. Kawashima, Viesturs Simanis, Masayuki Yamamoto, Masamitsu Sato

**Affiliations:** 1 Department of Biophysics and Biochemistry, Graduate School of Science, The University of Tokyo, 7-3-1 Hongo, Tokyo 113-0033, Japan; 2 Graduate School of Pharmaceutical Sciences, The University of Tokyo, 7-3-1 Hongo, Tokyo 113-0033, Japan; 3 EPFL SV ISREC UPSIM SV2.1830, Station 19, Lausanne 1015, Switzerland; 4 Laboratory of Cell Responses, National Institute for Basic Biology, Nishigonaka 38, Myodaiji, Okazaki, Aichi 444-8585, Japan; 5 PRESTO, Japan Science and Technology Agency, Gobancho, Chiyoda-ku, Tokyo 102-0076, Japan; 6 Laboratory of Cytoskeletal Logistics, Department of Life Science and Medical Bioscience, Graduate School of Advanced Science and Engineering, Waseda University, TWIns, 2-2 Wakamatsucho, Shinjuku, Tokyo 162-8480, Japan

**Keywords:** cell cycle, cyclin-dependent kinase, chemical genetics, analogue-sensitive mutant, fission yeast

## Abstract

Analogue-sensitive (as) mutants of kinases are widely used to selectively inhibit a single kinase with few off-target effects. The analogue-sensitive mutant *cdc2-as* of fission yeast (*Schizosaccharomyces pombe*) is a powerful tool to study the cell cycle, but the strain displays meiotic defects, and is sensitive to high and low temperature even in the absence of ATP-analogue inhibitors. This has limited the use of the strain for use in these settings. Here, we used *in vivo* selection for intragenic suppressor mutations of *cdc2-as* that restore full function in the absence of ATP-analogues. The *cdc2-asM17* underwent meiosis and produced viable spores to a similar degree to the wild-type strain. The suppressor mutation also rescued the sensitivity of the *cdc2-as* strain to high and low temperature, genotoxins and an anti-microtubule drug. We have used *cdc2-asM17* to show that Cdc2 activity is required to maintain the activity of the spindle assembly checkpoint. Furthermore, we also demonstrate that maintenance of the Shugoshin Sgo1 at meiotic centromeres does not require Cdc2 activity, whereas localization of the kinase aurora does. The modified *cdc2-asM17* allele can be thus used to analyse many aspects of cell-cycle-related events in fission yeast.

## 2. Introduction

Phosphorylation is involved in many cellular events, often serving as a molecular switch to regulate signalling pathways. The fission yeast genome contains 96 protein kinases (www.pombase.org [1]). A variety of genetic materials and methods have been developed to investigate the function of each kinase in *Schizosaccharomyces pombe*. For kinases that are indispensable for cell growth, it is common to use conditional mutants to knockdown the gene function. Those include temperature-sensitive (ts) mutants, which lose functions at the restrictive temperature. Although this method provides a powerful genetic tool, it poses practical problems for cell biology. For instance, it is difficult to reduce kinase activity rapidly during live-cell imaging because of the technical difficulties involved in changing the temperature. Furthermore, most mutants are not well characterized with regard to how fast the activity is lost following shift to the restrictive temperature. Small molecules are frequently used as kinase inhibitors, particularly for the analysis of cultured mammalian cells; however, many of these work poorly on yeast cells (for example, fig. 1 of [2]).

These technical difficulties were solved by a so-called chemical genetics approach [3]. Substitution of a single amino acid in the adenosine triphosphate (ATP)-binding pocket of a kinase (the so-called gatekeeper residue) renders the mutant kinase sensitive to ATP-analogue molecules that cannot fit into the active site of an unmodified kinase. This confers specificity to the inhibitor, as genetically unmodified kinases are unaffected by ATP-analogues. They also inhibit the kinase function rapidly (approximately minutes after a drug addition to the medium [4]), permitting time-lapse observation over short time scales. In principle, the gatekeeper residue of any kinase can be predicted from its amino acid sequence [5,6], which has prompted the construction of an as-mutant collection of fission yeast essential kinases [7]. Analogue-sensitive mutants are now widely used for analyses of cell cycle regulation.

Mitotic progression is controlled by protein kinases that have been conserved from yeast to human [8]. The main mitotic kinases include cyclin-dependent kinase 1 (Cdk1), known as Cdc2 in fission yeast and Cdc28 in budding yeast [9–11]; the polo kinase, known as Plo1 in fission yeast and Cdc5 in budding yeast [12–14]; and the single aurora kinase (equivalent to aurora B), which is known as Ark1 in fission yeast and Ipl1 in budding yeast [15,16]. Analogue-sensitive mutants of these mitotic kinases have been described: fission yeast *cdc2-as* [17] and budding yeast *cdc28-as1* [3] for Cdk1; *cdc5-as* [18] and *plo1-as* [7] for polo kinase; and *ark1-as2*/*as3* [19] and *ipl1-as* [5] for aurora B kinase.

Cdc2/Cdc28 regulates both the G1/S and G2/M transitions in *S. pombe* and *Saccharomyces cerevisiae*. Comprehensive proteomics analyses using *cdc28-as1* in budding yeast have identified more than 300 Cdk1 substrates [4,20]. In *S. pombe*, Cdc2 is required both for the G1/S and G2/M transitions (for review, see [21]). Many mitotic substrates have been identified; for example, Cdc2 is required for activation of Plo1 [22], for faithful chromosome segregation through controlling localization of the chromosomal passenger complex (CPC) [23] and Nsk1 [24], for chromosome condensation through nuclear accumulation of the condensin Cut3/SMC4 [25,26], and for localization of the microtubule-associated protein (MAP) Dis1/tumour overexpressed gene (TOG) at kinetochores [27,28]. Cdc2 is also required for activation of the anaphase-promoting complex (APC/cyclosome) [29–31].

Combining analogue-sensitive mutants of mitotic kinases with microscopy of living cells provides a way to investigate kinase function during short periods of the cell cycle (e.g. metaphase or anaphase): addition of the inhibitory analogue decreases the activity of the kinase rapidly, to the extent desired [17,32]. This approach has revealed that Cdc2 is required for the nuclear accumulation of the MAP complex Alp7/transforming acidic coiled-coil-Alp14/TOG in early stages of mitosis, which facilitates bipolar spindle assembly [33–35], and in late mitosis, downregulation of Cdc2 promotes the asymmetric localization of septum initiation network proteins at spindle pole bodies (SPBs, the centrosome equivalent in yeast) [17,36].

Although analogue-sensitive mutants can be easily made by substitution of the gatekeeper amino acid, this mutation occasionally has deleterious effects. The fission yeast *cdc2-as* mutant is generated by the F84G mutation. However, in addition to analogue sensitivity, the cells are elongated at 25°C, indicating a delay in mitotic commitment, and they are also heat-sensitive, particularly at 36°C, even in the absence of the ATP-analogue molecule [17]. In addition, the *cdc2-as* mutant is defective in sexual differentiation (mating and meiosis) and the mutant zygotes produce two-spore asci instead of four-spore ones of the wild-type zygotes [17]. Such phenotypes have been observed in other hypomorphic alleles of Cdc2 [37–39] and the meiotic mutant *cdc2-N22/tws1* [40,41], indicating that the gatekeeper mutation reduces Cdc2–Cdc13 activity *per se*. This limits the usefulness of the *cdc2-as* allele in certain circumstances. For example, it is difficult to combine *cdc2-as* with many heat-sensitive mutants that require incubation at 36°C to arrest efficiently (e.g. *cdc25–22* [42]); *cdc2-as* also shows a negative interaction with mutants that arrest in mitosis, such as the β-tubulin mutants *nda3-311* (cs) and *nda3/alp12-1828* (ts) [43–45]. This incompatibility of the *cdc2-as* mutation with key mutants used to impose cell cycle arrests limits its utility for the analysis of some mitotic functions of Cdc2–Cdc13.

Meiosis in fission yeast consists of pre-meiotic DNA replication, meiotic recombination during meiotic prophase, and two consecutive rounds of chromosome segregation (meiosis I and meiosis II) without an intervening S phase, prior to sporulation. To achieve this meiosis-specific cell cycle progression, the Cdc2 activity is regulated in a special manner during meiosis. A fraction of Cdc13/cyclin B is protected from degradation even after anaphase onset of meiosis I to provide CDK activity for the onset of meiosis II [46]; this contrasts with the situation in mitosis, where Cdc13 is entirely degraded at anaphase onset. Degradation of Cdc13 by the APC/cyclosome is inhibited by Mes1 after anaphase onset of meiosis I [46], whereas CDK must be fully inactivated after meiosis II to avoid ‘meiosis III’ [47]. The unique modulation of Cdc2–Cdc13 implies that the function of Cdc2 in meiosis may differ from that in mitosis. The multiple roles of Cdc2 in meiosis have limited the use of conditional mutants to analyse its function. The existing *cdc2-as* mutant is also limited in its suitability for studies in meiosis, owing to production of dyads, in contrast to tetrads that wild-type zygotes produce [17].

Thus, although the previously described *cdc2-as* mutant is a powerful tool, it also has technical limitations in some experimental settings. We therefore decided to use natural selection to modify the *cdc2-as* allele to eliminate the undesirable hypomorphic phenotypes by additional mutations. We have used this improved *cdc2-as* allele to examine various functions of *cdc2* during mitosis and meiosis.

## 3. Results and discussion

### 3.1. Isolation of intragenic suppressor mutants of *cdc2-as*

**Figure 1.**
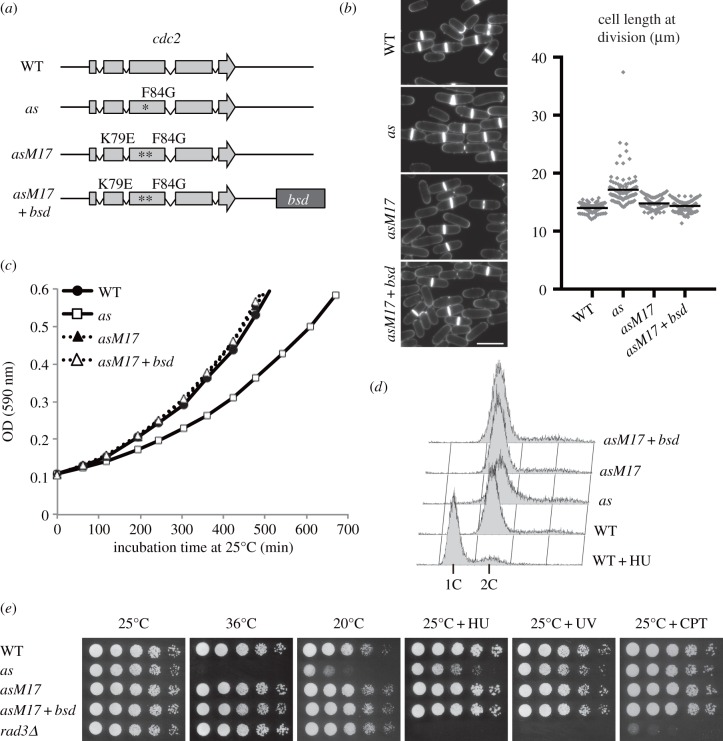
Characterization of the *cdc2-asM17* mutant in mitotic cell cycle. (*a*) Schematic of wild-type *cdc2* (WT), *cdc2-as* (*as*), *cdc2-asM17* (*asM17*) and *cdc2-asM17 + bsd* (*asM17 + bsd*) mutant genes. The *bsd* marker was inserted in the downstream of the *cdc2* coding sequence. (*b*) Calcofluor staining of vegetative cells at 25°C. Scale bar, 10 µm. The scatter-dot plot indicates distribution of cell length at cell division (µm; *n* ≥ 100). Black bars indicate mean values (mean ± s.e.: WT = 14.0 ± 0.1, *as* = 17.2 ± 0.3, *asM17* = 14.8 ± 0.1, *asM17 + bsd* = 14.3 ± 0.1). (*c*) OD (590 nm) measurement of log-phase cultures at 25°C. (*d*) FACS results showing the DNA content of vegetative cells at 25°C. For control of 1C DNA content, 12 mM HU was added to the WT culture (WT + HU). (*e*) Fivefold dilutions of the indicated strains were spotted onto the following media: YE containing 5 mM HU or 5 µM CPT, YE irradiated with 100 J m^−2^ UV. Plates were incubated at the indicated temperature for 3–6 days.

The original *cdc2-as* mutant gene contains a single amino acid substitution (F84G) at the gatekeeper residue [17] (figure 1*a*). We performed an error-prone PCR to introduce additional mutations to the *cdc2-as* gene containing the open reading frame and 500 bp upstream and downstream flanking regions (the strategy is summarized in the electronic supplementary material, figure S1*a*). The amplified 2.2 kb fragment was used for transformation of the original *cdc2-as* mutant, selecting for colony formation at 36°C (electronic supplementary material, figure S1*a,b*). We expected that these survivors should include intragenic suppressor mutations. We chose 17 colonies that survived at 36°C, and restreaked onto the YE5S plate (rich medium) containing phloxine B, which stains dead cells. Eight of 17 colonies (named M1, M2, M6, M8, M10, M11, M12 and M17) grew well at 36°C as well as at 20°C (electronic supplementary material, figure S1*b*), therefore those candidates were neither ts nor cs. Importantly, all of these survivors remained sensitive to the ATP-analogue molecule 1NM-PP1 (electronic supplementary material, figure S1*b*), indicating that none of the survivors were revertants of the as mutation.

Based upon these assays, we retained the M17 mutant, in which ts and cs phenotypes were significantly suppressed, for further analyses (the mutant allele is called *cdc2-asM17* hereafter; figure 1*a*). We next inserted the *bsd* gene (conferring the blasticidin S resistance [48]) as the selection marker for the modified *cdc2-asM17* gene. The *bsd* gene was inserted at the approximately 0.5 kb downstream of the termination codon of the *cdc2-asM17* gene (the allele is called *cdc2-asM17 + bsd* hereafter; figure 1*a*), and this did not affect the function of Cdc2 (electronic supplementary material, figure S1*b,c*).

To validate *cdc2-asM17* and *cdc2-asM17 + bsd* mutants as new tools for general purposes, we evaluated whether they behave normally in the absence of ATP-analogues, because the original *cdc2-as* mutant is slightly deficient in cell cycle [17]. First, we measured the cell size of *cdc2-asM17* (±*bsd*) mutants at cell division. As shown in figure 1*b*, *cdc2-as* cells were slightly longer than wild-type (WT) cells [17], indicating compromised CDK activity. The elongation was not observed in the *cdc2-asM17* and *cdc2-asM17 + bsd* strains. This was confirmed by a growth curve assay of four strains (WT, *cdc2-as*, *-asM17*, *-asM17 + bsd*; figure 1*c*): the growth of the *cdc2-as* strain was slightly slower than WT, whereas the *cdc2-asM17* and *-asM17 + bsd* strains grew at the same rate as WT. Next, we performed FACS analysis to examine the DNA content of *cdc2-asM17* mutants. As shown in figure 1*d*, *cdc2-asM17* and -*asM17 + bsd* mutants displayed similar DNA content profiles compared with WT, indicating that cell cycle progression of those mutants is similar to WT in the absence of the inhibitory drug. Because the DNA structure check point depends upon Cdc2 for activity, we examined whether *cdc2-asM17* mutants were sensitive to genotoxins. Although the original *cdc2-as* strain was slightly sensitive to hydroxyurea (HU), UV and camptothecin (CPT), the *cdc2-asM17* strains were not (figure 1*e*). Finally, we investigated whether *cdc2-asM17* remains associated with Suc1 (p13suc1), which is known to interact with the Cdc2-cyclin B complex [49]. A pulldown assay using p13 Suc1-beads indicated that the *cdc2-asM17* mutation did not affect interaction between CDK and Suc1, in the absence of ATP-analogues (electronic supplementary material, figure S2).

Together the data described above, we demonstrate that the *cdc2-asM17* mutant (±*bsd*) behaves similarly to WT in assays where the original *cdc2-as* allele shows clear defects, thereby validating its use to study the role of Cdc2 activity during mitotic cell cycle in several conditions where the original *cdc2-as* was not functional. Insertion of the *bsd* marker gene at the downstream of the *cdc2* gene did not cause abnormality as far as we have tested (figure 1*b–e*).

**Figure 2.**
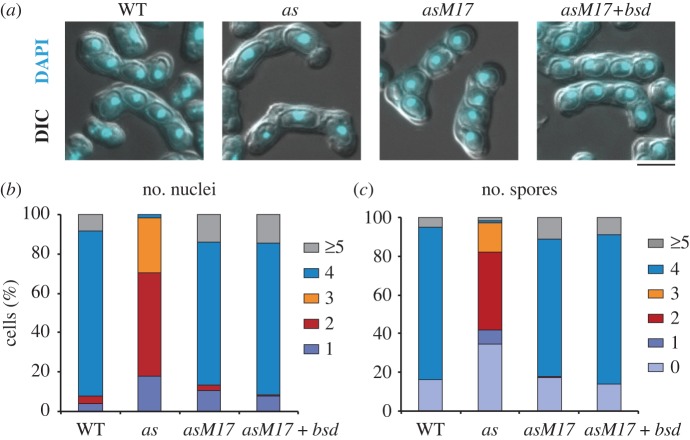
Characterization of the *cdc2-asM17* mutant in meiosis. (*a*) DAPI staining of cells that underwent mating, meiosis and sporulation. DAPI and differential interference contrast (DIC) images are shown merged. Scale bar, 5 µm. (*b,c*) The numbers of (*b*) nuclei and (*c*) spores in each ascus shown in (*a*) were counted for the indicated strains and the percentages are shown. (*n* > 200).

Next, we investigated if the *cdc2-asM17* mutant suppressed the meiotic defects seen in the original *cdc2-as* mutant. Homothallic *h^90^ cdc2-asM17* cells underwent mating and meiosis, and mostly generated four nuclei and four spores per cell, as in WT, whereas the original *cdc2-as* cells generated abnormal two or three nuclei and two or three spores [17] (figure 2*a–c*). *cdc2-asM17* + *bsd* also generated four nuclei and four spores per cell (figure 2*a–c*). Spore viabilities of *cdc2-asM17* (91%, *n* = 108) and *cdc2-asM17* + *bsd* (94%, *n* = 108) are comparable with that of WT (100%, *n* = 104), indicating that *cdc2-asM17* and *cdc2-asM17 + bsd* mutants undergo meiosis and produce viable spores to a similar extent to WT. Thus, these mutants are suitable for analysis of Cdc2 functions in meiosis, which could not be addressed using the original *cdc2-as* mutant. Sporulation of five other suppressor mutants (M1–M11) was also examined, and sporulation defects of the original *cdc2-as* mutant were in general suppressed to some extent (electronic supplementary material, figure S3).

### 3.2. Characterization of the suppressor mutations

**Figure 3.**
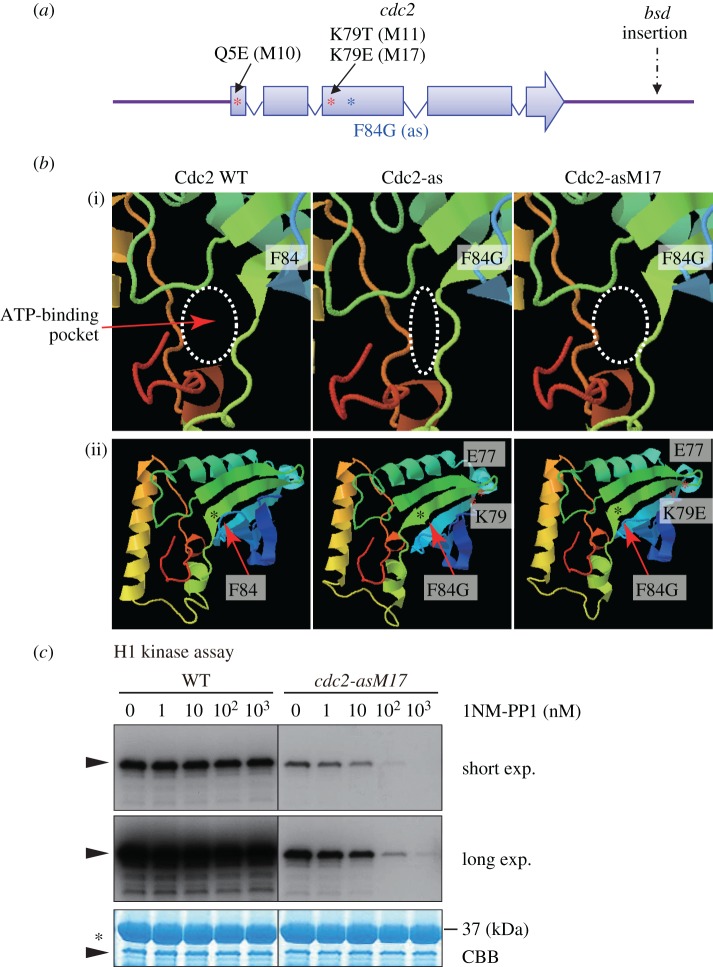
Intragenic suppressor mutations of the isolated *cdc2* mutants. (*a*) The schematic of the *cdc2* gene. The analogue-sensitive mutation is F84G [17]. The suppressor mutant M10 contained the Q5E substitution. The M11 and M17 mutants shared the mutation site K79 to T (M11) and to E (M17) in the open reading frame of the *cdc2* gene, in addition to the F84G mutation. (*b*) The prediction of secondary and tertiary structure of Cdc2 WT (wild-type), Cdc2-as and Cdc2-asM17 proteins made by the Phyre program. (i) Magnified view around the ATP-binding pocket and the gatekeeper residue F84 (or F84G). (ii) The overview for (i). The mutation site K79E and a reference site E77 are shown with red asterisks, and F84G is shown with black asterisks. (*c*) *In vitro* kinase assay using Cdc2 WT and Cdc2-asM17 proteins purified from WT and the mutant strains, respectively. The Cdk1 substrate histone H1 was incubated with purified Cdc2 proteins and [^32^P]-α-ATP in the indicated concentration of 1NM-PP1. Autoradiograph images with a short and long exposure are shown. CBB; Coomassie brilliant blue straining for the loading control. Arrowheads indicate the position of histone H1, and the asterisk band corresponds to GST-tagged p13Suc1 derived from Suc1-beads used in affinity purification.

We then sequenced the *cdc2* gene of the suppressor mutants. Interestingly, mutants M11 and M17 possessed mutations at the same residue, lysine 79 (figure 3*a*). M11 had a substitution of K79 to T (threonine), whereas M17 had a substitution of K79 to E (glutamic acid). The mutation site was close to the as mutation site F84G in the primary structure. M10 had a substitution of glutamine at five to glutamic acid (Q5E). To explore how the suppressor mutation in the mutant M17 (K79E) suppressed the as mutation (F84G), the secondary and tertiary structure of the mutant protein was subjected to the protein folding prediction program Phyre2 (http://www.sbg.bio.ic.ac.uk/phyre2/) [50]. For the sake of simplicity, we used amino acid residues 1–149 (from the N-terminus to the β7 sheet), which form the N-terminal lobe of the kinase [51]. Introduction of the analogue-sensitive mutation F84G was predicted to generate a structural alteration around the ATP binding pocket (Cdc2-as; figure 3*b*). The deformation of the ATP binding pocket was suppressed by the additional introduction of K79E (Cdc2-asM17; figure 3*b*). Consistent with this result, mutations within a β sheet in the N-terminal lobe have been reported to suppress the gatekeeper mutations that are not tolerated in several kinases [52]. It is possible that the introduction of K79E, which locates at the edge of the β sheet, may twist the sheet, so that the opposite edge of the sheet harbouring F84G alters the angle at the ATP-binding pocket. Kinase assays of WT Cdc2 and Cdc2-asM17 revealed that the activity of asM17, but not of WT Cdc2, was inhibited by addition of 10^2^–10^3^ nM of the ATP-analogue 1NM-PP1 (figure 3*c*). Although the suppressor mutation rescues all the phenotypic defects of *cdc2-as*, the *in vitro* kinase assay nonetheless reveals that Cdc2-asM17 is less active than WT Cdc2 in the absence of 1NM-PP1 (figure 3*c*).

### 3.3. Cdc2 and the SAC: the use of *cdc2-asM17* with MBC or the β-tubulin ts mutant

Elevation of the Cdc2 kinase activity induces assembly of the bipolar spindle at mitotic onset. If microtubule formation is perturbed by internal mutations or drugs, then the attachment of kinetochores to microtubules may be inhibited. The spindle assembly checkpoint (SAC) monitors kinetochore–microtubule attachment to ensure faithful chromosome segregation in mitosis. The SAC components Mad1–Mad2 recognize unattached kinetochores and arrest cell cycle progression at metaphase until correct attachment has been accomplished. Recent studies in yeast, flies, frogs and humans have suggested that Cdk1 can serve as an upstream activator of the SAC [53–58]. We therefore used the *cdc2-asM17* mutant to examine whether CDK activity is required to maintain an SAC arrest in *S. pombe*.

**Figure 4.**
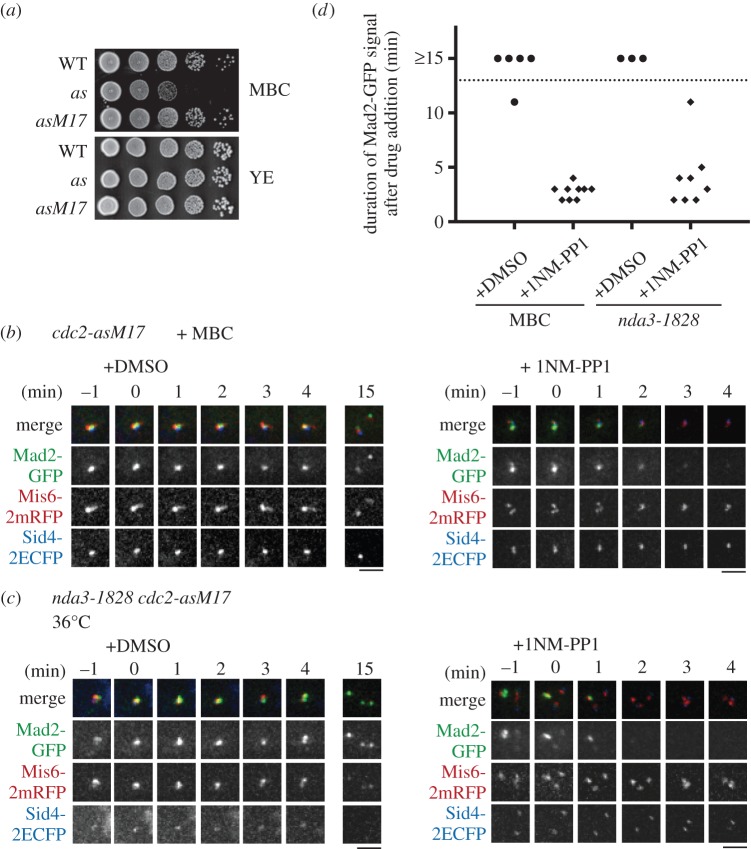
The requirement of Cdc2 in SAC maintenance was revealed by use of the *cdc2-asM17* mutant. (*a*) The original *cdc2-as* mutant (*as*) was sensitive to a low dose of the microtubule drug MBC (10 µg ml^−1^), whereas the revived mutant *cdc2-asM17* (*asM17*) was not. This enabled use of the analogue-sensitive *cdc2* mutant in the presence of MBC. (*b*) The c*dc2-asM17* mutant was used in combination with MBC treatment. *cdc2-asM17* cells were treated with MBC to arrest cells at metaphase without spindles. Cells with Mad2-GFP dots at kinetochores were chosen for filming. After 1NM-PP1 addition (*t* = 0 min), Mad2-GFP dots disappeared within 4 min. DMSO was added as negative control. Mis6-2mRFP, a kinetochore marker; Sid4-2ECFP, an SPB marker. (*c*) The *cdc2-asM17* mutation was combined with the β-tubulin *ts* mutation *nda3/alp12–1828*. Cells were arrested at metaphase at 36°C. Filming was done similarly to (*b*). After 1NM-PP1 addition (*t* = 0 min), Mad2-GFP dots disappeared within 3 min. DMSO was added as negative control. Scale bars, 2 µm. (*d*) Duration of Mad2-GFP dots residence at kinetochores after addition of DMSO or 1NM-PP1 in (*b,c*) was measured.

First, we tested whether the microtubule drug methyl benzimidazol-2-yl-carbamate (MBC) induces *cdc2-as* cells to arrest at metaphase; the original *cdc2-as* mutant was sensitive to a low concentration of MBC (10 µg ml^−1^), which did not prevent colony formation in WT cells (figure 4*a*). By contrast, the *cdc2-asM17* mutant was not sensitive to MBC (figure 4*a*). We therefore constructed the *cdc2-asM17* strain expressing Mad2-GFP, the kinetochore marker Mis6-2mRFP and the SPB marker Sid4-2ECFP. Cells were treated with MBC to induce metaphase arrest; bright Mad2-GFP signal was observed at kinetochores, which is a hallmark of SAC activation (−1 min, figure 4*b*). Cells were then filmed and 1NM-PP1 or DMSO was added to the medium (0 min, figure 4*b*). In the DMSO treatment, Mad2-GFP dots remained at kinetochores for longer than 15 min (+DMSO, figure 4*b,d*). By contrast, Mad2-GFP dots mostly disappeared within 4 min after 1NM-PP1 addition (+1NM-PP1, figure 4*b,d*). This demonstrates that Cdc2 kinase activity is required for maintenance of SAC activation in the presence of microtubule perturbation, through Mad2 recruitment to kinetochores. A very recent study in human cells has shown that Mad2 recruitment to kinetochore requires Cdk1 activity [59], suggesting that this mechanism has been conserved through evolution.

We examined this further using the ts β-tubulin mutant *nda3/alp12-1828*, which was impossible to combine with the original *cdc2-as* mutant, because the long G2-phase of *S. pombe* results in a predominant G2-arrest owing to temperature sensitivity of the *cdc2-as* allele. After shift to 36°C, the *nda3-1828 cdc2-asM17* mutant efficiently arrested at prometaphase without a spindle, and Mad2-GFP dots were associated with kinetochores (−1 min, figure 4*c*). In the presence of 1NM-PP1, Mad2-GFP dots mostly disappeared within 4 min (+1NM-PP1, figure 4*c,d*), confirming that SAC maintenance requires Cdc2 activity during mitosis. These experiments also demonstrate the utility of the analogue-sensitive mutant *cdc2-asM17* to investigate the involvement of Cdc2 in mitotic events such as the SAC.

### 3.4. SAC silencing and PP1: the use of *cdc2-asM17* with the β-tubulin cs mutant

**Figure 5.**
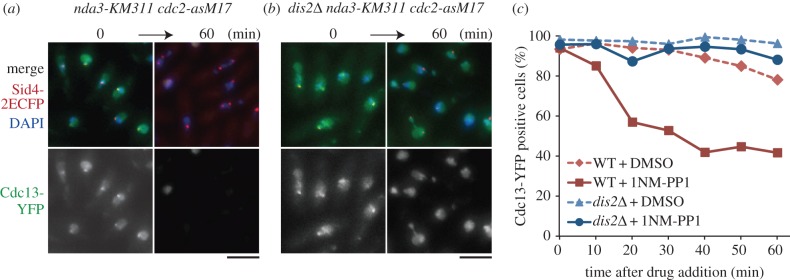
Dis2/PP1 is required for checkpoint inactivation triggered by Cdc2 inhibition. (*a*) The *cdc2-asM17* mutation was combined with the *cs* β-tubulin mutation *nda3-KM311*. The mutant was arrested at metaphase at 18°C with high concentration of cyclin B1/Cdc13-YFP in the nucleus and at SPBs (0 min). Then, 1NM-PP1 was added to the culture. After 60 min, the percentage of cells with Cdc13-YFP decreased. Sid4–2ECFP (SPB) and DAPI are also shown. (*b*) Similar experiments were done with the triple mutant *dis2*Δ *nda3-KM311 cdc2-asM17*. Cells were cultured at 18°C (0 min) and then 1NM-PP1 was added. In contrast to *dis2*^+^ cells (*a*), Cdc13-YFP remained in the nucleus. Scale bars, 5 µm. (*c*) Frequencies of cells with nuclear Cdc13-YFP signal in *dis2*^+^ (WT) and *dis2*Δ strains in the *nda3-KM311 cdc2-asM17* background were plotted in response to 1NM-PP1 (or DMSO for negative control) addition (*n* ≥ 100).

Recently, it was reported that SAC silencing (inactivation) during mitosis is achieved by the protein phosphatase PP1 [60–63]. The fission yeast has two PP1 phosphatases, Dis2 and Sds21 [64,65], though only Dis2 has a function in SAC silencing [61]. To test whether Dis2/PP1 is required for the SAC silencing following Cdc2 inactivation (as shown in figure 4), we created the double mutant *cdc2-asM17 nda3-KM311*. The double mutant of the original *cdc2-as* and *nda3-KM311* did not enter mitosis efficiently, owing to the cold-sensitivity of *cdc2-as* [17]. By contrast, the *cdc2-asM17 nda3-KM311* cells arrested efficiently in mitosis at 18°C with a strong nuclear signal of cyclin B/Cdc13-YFP, (0 min, figure 5*a,c*). When 1NM-PP1 was added to the medium, the frequency of cells harbouring Cdc13-YFP signal decreased (60 min, figure 5*a,c*). In contrast, when the *dis2*^+^ gene was disrupted, the triple mutant *dis2*Δ *nda3-KM311 cdc2-asM17* retained a Cdc13-YFP signal even after addition of 1NM-PP1 (figure 5*b,c*). This indicates that Cdc2 and Dis2/PP1 are required to maintain and silence the checkpoint machinery, respectively. Ark1/aurora B kinase, a component of the CPC, is also required for SAC maintenance [61]. As centromere targeting of CPC depends on Cdk1 [23], maintenance of the SAC by Cdc2 might be achieved through the control of CPC localization to centromeres. Alternatively, Cdc2 might regulate the SAC independently of CPC localization, because centromeric retention of CPC in anaphase does not result in APC/C inhibition (SAC activation) in human cells [66]. How Dis2 /PP1 counteracts Cdc2 in SAC silencing is an important question and will be addressed in future studies. The *dis2*Δ *nda3-KM311 cdc2-asM17* mutant frequently resulted in unequal chromosome segregation with persistent nuclear Cdc13-YFP (figure 5*b*), indicating that checkpoint adaptation (slippage into anaphase) may occur even without complete cyclin destruction, when the Cdc2 activity is inhibited.

### 3.5. Shugoshin and Cdc2: the use of *cdc2-asM17* in meiosis

Genetic perturbation of Cdc2 prevents entry into meiosis I [67], making it difficult to examine the function of Cdc2 in spindle organization and chromosome segregation during meiosis I. Because the *cdc2-asM17* strain does not show meiotic defects in the absence of ATP-analogues we used it to address this question.

We have previously used *cdc2-asM17* strain to show that reorganization of SPBs at the onset of meiosis I requires Cdc2 activity [68]. We have now used it to examine the role of Cdc2 activity at the metaphase–anaphase transition in meiosis I. To ensure faithful segregation of chromosomes during meiosis I, proteins such as aurora B and protein phosphatase 2A (PP2A) are localized transiently to centromeres, until the onset of anaphase I [69,70]. It is unclear whether delocalization of aurora B and PP2A at the onset of anaphase I depends on reduction of the Cdc2 activity or proteasome-dependent protein degradation.

**Figure 6.**
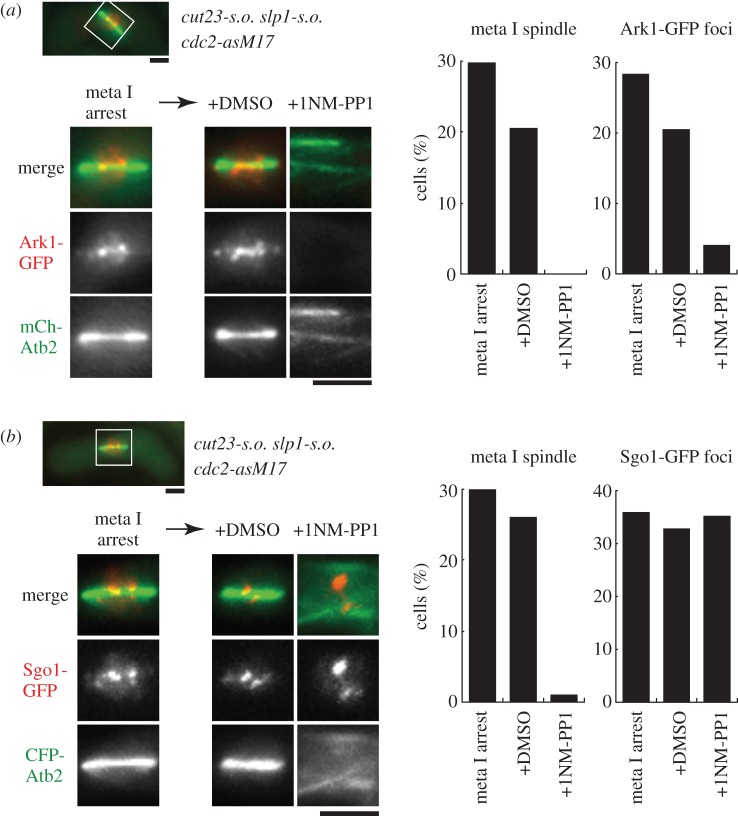
Cdc2 activity is required for aurora-B localization but not for the Shugoshin Sgo1 during meiosis I. (*a*) The *cdc2-asM17* mutation was combined with *slp1-s.o.* and *cut23-s.o.* mutations, in which meiotic transcription of these APC/C factors is shut off and cells are arrested in metaphase I. The rectangular region was magnified and is shown below. The aurora B/Ark1-GFP localized to centromeres on the metaphase spindle, which was visualized by CFP-Atb2 (α2-tubulin). Ark1-GFP foci dispersed after 1NM-PP1 treatment, but not after DMSO treatment (negative control). Images corresponding to the nuclear region were taken 1 h after addition of 1NM-PP1 or DMSO. The frequencies of cells with the metaphase I spindle (the left graph) and with Ark1-GFP foci (the right graph) are also shown (*n* > 100). (*b*) Similar experiments to (*a*) were done for Sgo1-GFP. Sgo1-GFP foci at centromeres did not disperse even 1 h after 1NM-PP1 addition (*n* > 100). Scale bars, 2 µm.

To distinguish these two possibilities, we used *slp1-s.o. cut23-s.o.* mutations, in which meiotic expression of the APC/C component Cut23 and its activator Slp1 is repressed [71], thereby preventing proteasome-dependent protein degradation. Then, 1NM-PP1 was added to inhibit Cdc2 activity of *cdc2-asM17* cells. First, we examined localization of Ark1 (aurora B). Cdc2 phosphorylates the CPC component Bir1/survivin to recruit Ark1 to centromeres in fission yeast mitosis [23], but it has not been tested whether this mechanism is conserved during meiosis. Ark1 localized to centromeres in cells arrested in metaphase I, and delocalized from centromeres when Cdc2 activity was reduced by addition of 1NM-PP1 (figure 6*a*). This indicates that delocalization of Ark1 from centromeres depends on reduction of the Cdc2 activity, but not on degradation of CPC components. Second, we examined the localization of Shugoshin (Sgo1), which forms a complex with PP2A and is essential for its localization at centromeres [70]. Consistent with previous studies [69], Sgo1 localized to centromeres in metaphase I-arrested cells. Interestingly, Sgo1 did not alter its localization at centromeres even when Cdc2 activity was decreased by 1NM-PP1 (figure 6*b*). Thus, maintenance of the Sgo1 localization at centromeres does not require Cdc2 activity, raising the possibility that APC-mediated Sgo1 degradation might be a trigger of Sgo1 delocalization from centromeres.

## 4. Conclusion

The generation of an analogue-sensitive mutant is a powerful tool that enables chemical inhibition of any kinase of interest. In general, analogue sensitivity is conferred by introducing a single amino acid substitution of the ‘gatekeeper’ residue in the active site. This sometimes causes a partial loss of kinase function, as in the case of the *cdc2-as* mutant [17]. The original *cdc2-as* mutant (F84G) showed defects in growth at high and low temperature and in meiosis. We have used natural selection to suppress these defects, which occurred through an additional mutation at K79E, to generate the *cdc2-asM17* mutant.

A systematic generation of analogue-sensitive mutants of all essential kinases in fission yeast found that three (of 16) essential kinases could not be generated (Cdc7, Hsk1 and Sid1), because an introduction of the analogue-sensitive mutation caused a significant loss of function [7]. For instance, it was impossible to create the *sid1-as* mutant, because replacement of the wild-type *sid1*^+^ with the *sid1-as* mutant gene caused lethality (Y.A., S.A.K., V.S., M.Y. & M.S. 2011, unpublished data). Those issues, however, might be solved by applying the method described in this study. Specifically, if the gatekeeper mutation resides at the end of a β sheet, then the suppressor mutation could be introduced at the opposite end of the sheet (figure 3*a,b*). Alternatively, it might be more judicious to allow *in vivo* selection to generate the required mutant, as we have done here (electronic supplementary material, figure S1*a*). The library of mutagenized DNA fragments could be used for transformation of the existing ts (or other) mutant of the kinase. Transformants viable at the restrictive temperature should contain the suppressor mutation in addition to the gatekeeper mutation.

The *in vivo* selected *cdc2-asM17* mutant permitted us to undertake experiments that were not possible previously, such as using microtubule-depolymerizing drugs, high and low temperatures, and during meiosis. This mutant has revealed an important role of Cdc2/Cdk1 in SAC maintenance, and the dispensability of Cdc2/Cdk1 for localization of Shugoshin in meiosis. Although mitotic kinases represented by Cdk1, Polo and aurora regulate many aspects of mitosis and meiosis, the availability of this chemical genetic tool will allow us to increase our understanding of the role of Cdc2/CDK1.

## 5. Material and methods

### 5.1. Yeast genetics and general manipulations

The strains used in this study are listed in table 1. We used standard methods for fission yeast genetics, as described previously [72]. Tagging of a single copy of a fluorescent protein (GFP) at the C-terminus of genes, and insertion of the *bsd* marker gene, was performed using standard PCR-based methods [73,74]. Tagging of multiple tandem copies of fluorescent proteins (2mRFP and 2ECFP) at the C-terminus of genes was performed as previously described [34]. All the fluorescent protein-fused genes are expressed by the native promoter and the *adh* terminator [73], except for *cdc13-YFP*, *mCherry-atb2* and *CFP-atb2* fusion genes. The *cdc13-YFP* fusion gene is expressed by the *cdc13*^+^ promoter and terminator. The *mCherry-atb2* gene is expressed by the P*adh15* promoter and the *adh* terminator, and the *CFP-atb2* gene is expressed by the P*adh13* promoter and the *adh* terminator [75]. 1NM-PP1 (Calbiochem, CA) was added to media at the concentration of 2 µM. For figure 1*b*, living cells were stained with Calcofluor (Sigma, MO), and the cell length was measured on the ImageJ software (NIH). For the FACS analysis in figure 1*d*, the DNA content was measured using BD FACSCalibur (BD, NJ). For figure 2*a–c*, homothallic *h*^90^ cells were induced to mating, meiosis and sporulation on sporulation agar plates. After incubation for 24 h at 30°C, cells were fixed with methanol and stained with 4′,6-diamidino-2-phenylindole (DAPI; Wako Pure Chemicals, Japan), and the number of nuclei and spores in each ascus were counted. For the spore viability assay, spores were dissected by the micromanipulator (Singer Instruments, Somerset, UK), and the percentage of spores that formed colonies was calculated. The checkpoint silencing assay in figure 5 was performed as previously described [61]. The prometaphase-arrested *nda3-KM311* cells at 18°C were treated with 1 µM 1NM-PP1 or DMSO. Cells were collected at the indicated time point, fixed with methanol and stained with DAPI (Wako Pure Chemicals).

**Table 1.** Strains used in this study. The origin of the strains is this study, except for JY878 and SP5959 (our stock) and PY328 (a gift from Y. Watanabe).

strain	genotype	figures
MJ1172	*h*^90^ *sfi1-CFP-nat leu1–32 ura4-D18 ade6-M216*	1*b–e*, 2*a–c* and electronic supplementary material, figure S2
MJ1254	*h*^90^ *cdc2-as sfi1-CFP-nat leu1–32 ura4-D18 ade6-M216*	1*b–e*, 2*a–c* and electronic supplementary material, figures S1*a–c*, S2 and S3*a–c*
MJ1353	*h*^90^ *cdc2-asM17 sfi1-CFP leu1–32 ura4-D18 ade6-M216*	1*b–e*, 2*a–c* and electronic supplementary material, figures S1*b,c*, S2 and S3*a–c*
MJ1358	*h*^90^ *cdc2-asM17-bsd sfi1-CFP-nat leu1–32 ura4-D18 ade6-M216*	1*b–e*, 2*a–c*, 3*c*, 4*a* and electronic supplementary material, figures S1*c* and S2
PY328	*h*^90^ *rad3 ::LEU2^+^ leu1–32 ura4-D18 ade6-M210*	1*e*
SAK1	*h*^+^ *leu1–32 ura4-D18 ade6-M216*	3*c*
MJ1360	*h*^90^ *cdc2-as-bsd sfi1-CFP-nat leu1-32 ura4-D18 ade6-M216*	4*a* and electronic supplementary material, figure S1*c*
YA1843	*h^90^ cdc2­asM17­bsd mad2­GFP­kan mis6­2mRFP­hph sid4­2ECFP­nat leu1­32 ura4­D18*	4*b,d*
YA1829	*h^90^ cdc2­asM17­bsd nda3(alp12)­1828 mad2­GFP­kan mis6­2mRFP­hph sid4­2ECFP­nat leu1­32 ura4­D18 ade6­M216*	4*c,d*
YA1900	*h^90^ cdc2­asM17­bsd nda3­KM311 cdc13­YFP­Tcdc13­kan sid4­2ECFP­nat leu1­32 ura4­D18 ade6*	5*a,c*
YA1893	*h^90^ cdc2­asM17­bsd nda3­KM311 dis2::ura4+ cdc13­YFP­Tcdc13­kan sid4­2ECFP­nat leu1­32 ura4­D18 ade6*	5*b,c*
SAK422	*h*^90^ *cdc2-asM17-bsd ark1-GFPFH-kan Prad21-slp1-kan Prad21-cut23-kan z::Padh15-mcherry-atb2-nat leu1-32 ade6-M216*	6*a*
SAK420	*h*^90^ *cdc2-asM17-bsd sgo1+-flag-GFP par1--mCherry-hyg Prad21-slp1-kan Prad21-cut23-kan z::Padh13-CFP-atb2-nat leu1-32 ade6-M216*	6*b*
JY878	*h*^90^ *leu1-32 ura4-D18 ade6-M216*	electronic supplementary material, figures S1*b,c* and S3*a*
SP5959	*h*^–^ *cdc2-as ura4-D18*	electronic supplementary material, figure S1*a*
MJ1346	*h*^90^ *cdc2-asM1 sfi1-CFP-nat leu1-32 ura4-D18 ade6-M216*	electronic supplementary material, figure S1*b*
MJ1347	*h*^90^ *cdc2-asM2 sfi1-CFP-nat leu1-32 ura4-D18 ade6-M216*	electronic supplementary material, figure S1*b*
MJ1348	*h*^90^ *cdc2-asM6 sfi1-CFP-nat leu1-32 ura4-D18 ade6-M216*	electronic supplementary material, figures S1*b* and S3*a*
MJ1349	*h*^90^ *cdc2-asM8 sfi1-CFP-nat leu1-32 ura4-D18 ade6-M216*	electronic supplementary material, figures S1*b* and S3*a–c*
MJ1350	*h*^90^ *cdc2-asM10 sfi1-CFP-nat leu1-32 ura4-D18 ade6-M216*	electronic supplementary material, figures S1*b,c* and S3*a–c*
MJ1351	*h*^90^ *cdc2-asM11 sfi1-CFP-nat leu1-32 ura4-D18 ade6-M216*	electronic supplementary material, figures S1*b,c* and S3*a–c*
MJ1352	*h*^90^ *cdc2-asM12 sfi1-CFP-nat leu1-32 ura4-D18 ade6-M216*	electronic supplementary material, figure S1*b*
MJ1356	*h*^90^ *cdc2-asM10-bsd sfi1-CFP-nat leu1-32 ura4-D18 ade6-M216*	electronic supplementary material, figure S1*c*
MJ1357	*h*^90^ *cdc2-asM11-bsd sfi1-CFP-nat leu1-32 ura4-D18 ade6-M216*	electronic supplementary material, figure S1*c*
MJ1359	*h*^90^ *cdc2^+^-bsd leu1-32 ura4-D18 ade6-M216*	electronic supplementary material, figure S1*c*
SAK163	*h*^–^ *cdc2-33*	electronic supplementary material, figure S2
MO1893	*h*^90^ *cdc2-asM17-bsd leu1-32 ura4-D18 ade6-M216*	Methods

### 5.2. Creation of the *cdc2-asM17* mutant

The creation of the original *cdc2-as* mutant has been described previously [17]. A flow chart for the method to generate the *cdc2-asM17* mutant is depicted in the electronic supplementary material, figure S1*a*. Briefly, the genomic *cdc2-as* mutant gene was amplified from the original mutant [17]. The coding region of *cdc2-as* with flanking 0.5 kb up/downstream regions at both ends (in total 2.2 kb; electronic supplementary material, figure S1*a*) was amplified by a standard PCR method with PrimeSTAR HS DNA polymerase (Takara-Bio, Japan). The amplified fragment was gel-purified, and used as the template for the following error-prone PCR. To induce errors, thermal cycling was performed for 40 cycles using the Ex Taq polymerase (Takara-Bio) and the same pair of oligomers used for the first PCR. The amplified fragments were then used for transformation of the *cdc2-as sfi1-CFP-nat* strain MJ1254 (Sfi1-CFP is an SPB marker). Colonies that grew at 36°C were restreaked onto YE5S plates containing Phloxin B, to visualize suppression of temperature sensitivity. Cold sensitivity was also tested at 20°C. Confirmation of the analogue sensitivity was done with YE5S plates containing 10 µM 1NM-PP1. The *bsd* marker gene was inserted 528 bp downstream of the termination codon of the *cdc2* gene. For figures 3–6, the *cdc2-asM17* mutant with *bsd* insertion was used and denoted as *cdc2-asM17* for simplicity. The *sfi1-CFP* SPB marker was removed through backcrossing of the *cdc2-asM17-bsd sfi1-CFP-nat* strain (MJ1358) with a wild-type strain without markers, to yield MO1893.

### 5.3. Microscopy

Images in figures 1*b* and 2*a* were acquired using an Axio Imager.M2 fluorescence microscope and AxioVision software (Zeiss, Germany). Live-cell imaging methods for figure 4 were described previously [34]. Briefly, live-cell imaging was performed with the DeltaVision-SoftWoRx system (GE Healthcare, UK). Cultured cells were mounted on a glass-bottom dish (Matsunami, Japan) coated with lectin and filled with minimal medium. Serial section images along the *z*-axis were acquired and stacked using the ‘quick projection’ protocol in SoftWoRx. Temperature-sensitive strains were observed in a temperature-controlled chamber to maintain 36°C during observation. MBC (carbendazim; Sigma, MO) was added to liquid culture at the final concentration of 50 µg ml^−1^ [76]. 2 µM 1NM-PP1 or DMSO was added to liquid media during observation. Images in figure 5 and electronic supplementary material, figure S3*a* were acquired by an Axioplan 2 fluorescence microscope (Zeiss) and SlideBook software (Leeds Precision, UK). Images in figure 6 were taken as described previously [77].

### 5.4. *In vitro* kinase assay and Western blotting

*Schizosaccharomyces pombe* protein extract from wild-type and *cdc2-asM17* cells was prepared, and the Cdc2–Cdc13 complex was purified using Suc1-beads (Millipore, MA). The pulldowns containing Cdc2–Cdc13 were mixed with histone H1 (New England BioLabs, UK) as a substrate, in the absence or presence of 1NM-PP1 (0–10^3^ nM). Samples were subjected to SDS–PAGE, and the gel was stained with Coomassie brilliant blue followed by autoradiography. For Western blotting in electronic supplementary material, figure S2, extracts or pulldowns by Suc1-beads were subjected to SDS–PAGE. The following antibodies were used: anti-Cdc2 monoclonal (1 : 1000; a gift from Y. Watanabe) and anti-tubulin monoclonal TAT-1 (1 : 5000; a gift from K. Gull).

## Supplementary Material

Adobe PDF - rsob-14-0063-File008.pdf
