# Supplementary material for: Optimization of the analogue-sensitive Cdc2/Cdk1 mutant by in vivo selection eliminates physiological limitations to its use in cell cycle analysis
Source: Open Biol. 2014 Jul 2;4(7):140063. doi: 10.1098/rsob.140063 (PMC4118601; doi:10.1098/rsob.140063)
Supplement: Adobe PDF - rsob-14-0063-File008.pdf [file rsob140063supp1.pdf]

Supplementary data

Optimisation of the analog-sensitive Cdc2/Cdk1 mutant by in vivo selection eliminates physiological limitations to its use in cell cycle analysis

Yuki Aoi, Shigehiro A. Kawashima, Viesturs Simanis, Masayuki Yamamoto and Masamitsu Sato

Supplemental Figure S1

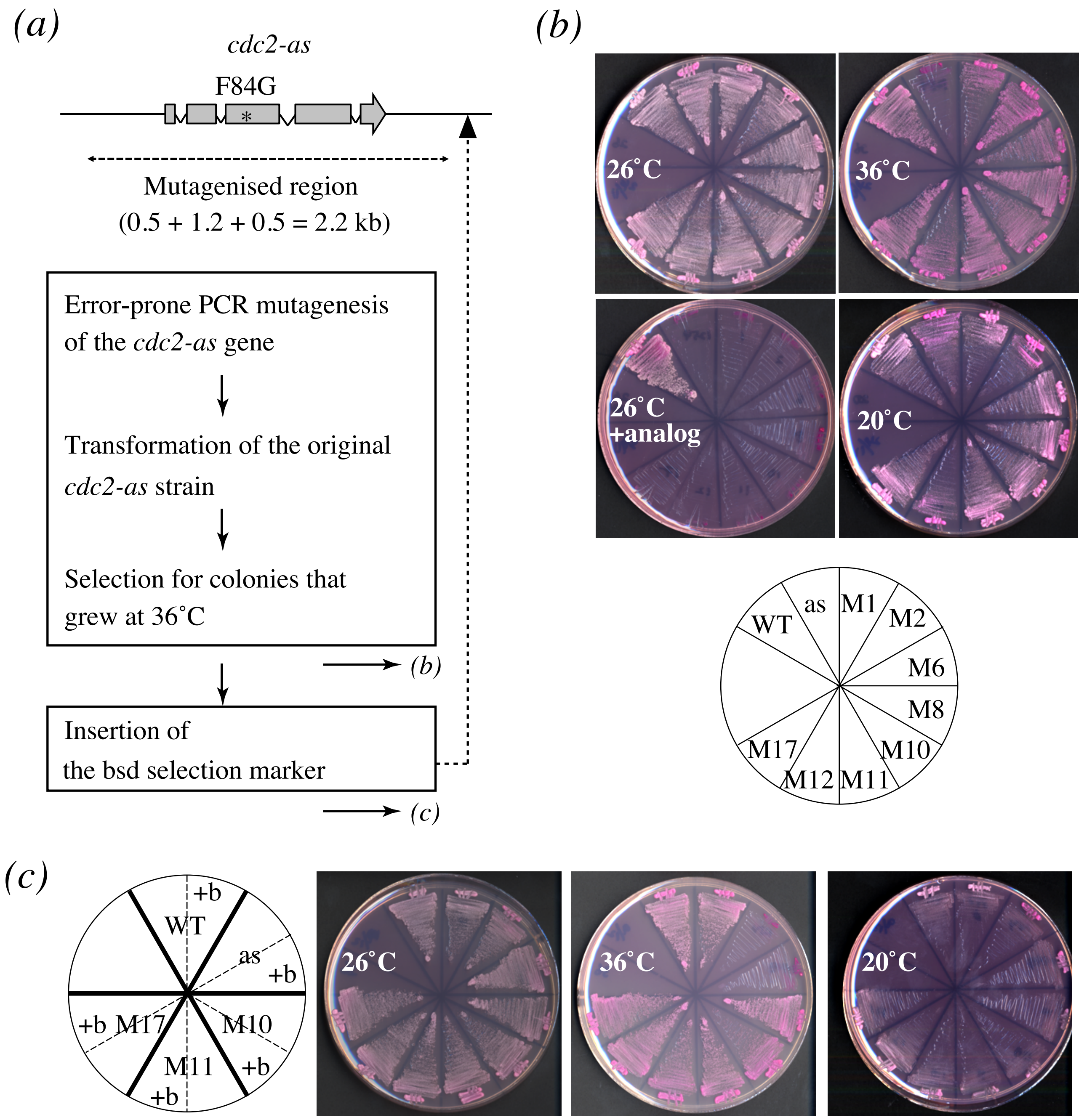

Supplemental figure 1. Isolation of suppressor mutants of *cdc2-as*

(a) A flowchart of the strategy to isolate the intragenic suppressor mutations for temperature sensitivity (*ts*) of the *cdc2-as* mutant. The DNA fragment containing the coding region (1.2 kb) and up- and down-stream (0.5 kb each) regions were amplified by standard PCR, followed by an error-prone thermal cycling, as described in materials and methods. The amplified fragments that contained random mutations were used for transformation of the *cdc2-as* mutant, and colonies were selected for the ability to grow at 36˚C, the restrictive temperature of the starting strain. Surviving colonies were restreaked onto plates to verify the *ts* and cold-sensitive (*cs*) suppression, as well as the 1NM-PP1 sensitivity (b). Then, the *bsd* selection marker was inserted at the 528 bp downstream of the *cdc2* gene; this did not affect the *ts/cs* suppression (c). (b)(c) Actual results during the screening procedures. (b) The candidate colonies (M1, M2, … M17) were restreaked onto the rich medium containing the red dye Phloxin B with or without 1NM-PP1. Plates were incubated at the indicated temperature. (c) The *bsd* marker gene was integrated into the three candidates (M10, M11, M17) as well as the wild-type (WT) and the original *cdc2-as* mutant. +b: strains with the *bsd* marker. Plates were incubated at the indicated temperature. Insertion of the *bsd* marker did not affect growth at least in the M17 mutant.

Supplemental Figure S2

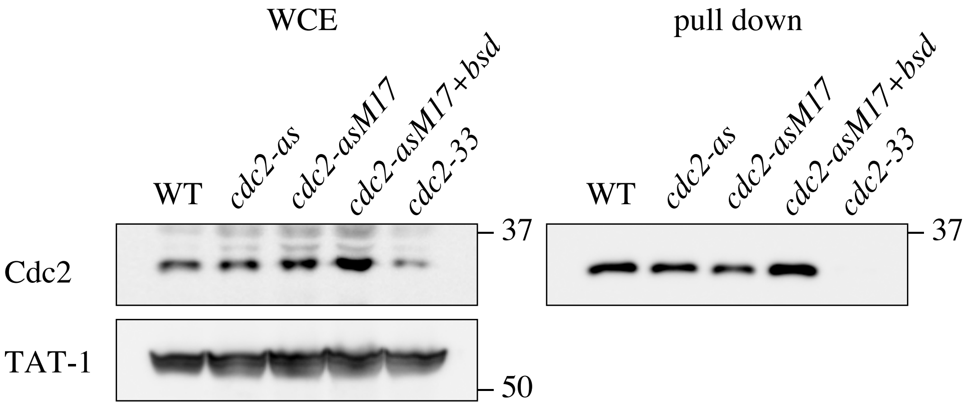

Supplemental figure 2. ***in vivo*** **interaction between Cdc2-asM17 and Suc1.**

Pull-down assay using Suc1-beads for the Cdc2 protein expressed in the indicated strain. The Cdc2-asM17 protein interacted with Suc1 to a similar extent of wild-type Cdc2 (WT). The *cdc2-33 ts* mutant was used as negative control for Suc1 interaction [1]. WCE: whole cell extract.

Supplemental Figure S3

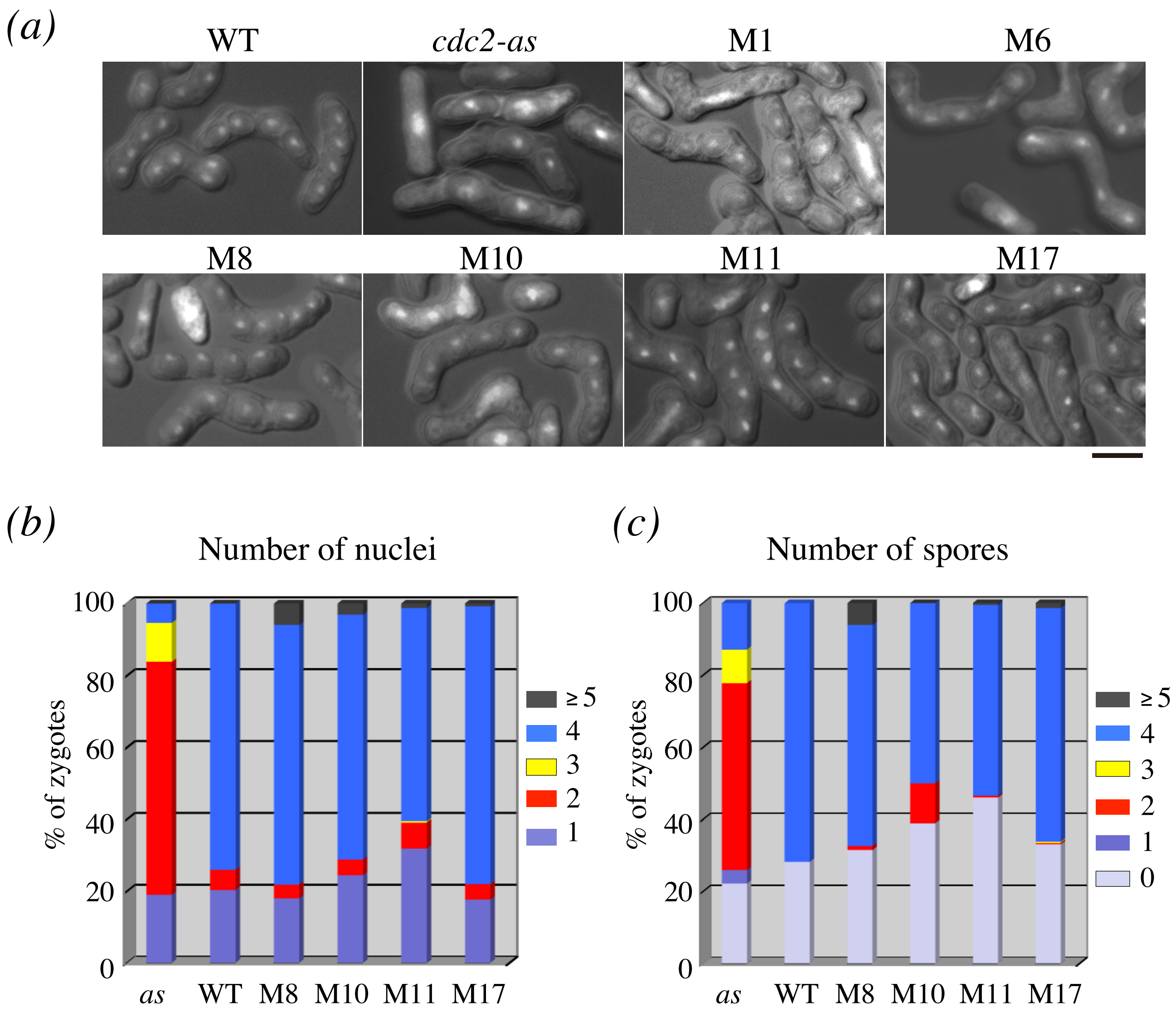

Supplemental figure 3. Meiotic defects were suppressed in the isolated mutants

(a) Mating, meiosis and sporulation were induced in WT, *cdc2-as* (original) and suppressor mutant candidates (M1~M17). The *cdc2-as* zygotes produced abnormal two-spored asci, whereas WT and others produced normal four-spored asci. DIC and DAPI images are shown merged. Scale bar, 5 μm. (b) The number of nuclei in each ascus in (a) was counted for the indicated strains, and the percentages are shown. (c) The number of spores in each ascus in (a) was counted and the percentages are shown. n = 74 (*cdc2-as*), 78 (WT), 184 (*cdc2-asM8*), 160 (*cdc2-asM10*), 236 (*cdc2-asM11*) and 256 (*cdc2-asM17*).

Reference for supplemental data

1. Booher, RN, Alfa, CE, Hyams, JS, and Beach, DH. 1989 The fission yeast cdc2/cdc13/suc1 protein kinase: regulation of catalytic activity and nuclear localization. *Cell* **58**, 485-497. (doi: http://dx.doi.org/10.1016/0092-8674(89)90429-7)
